# High-dimensional single cell mass cytometry analysis of the murine hematopoietic system reveals signatures induced by ageing and physiological pathogen challenges

**DOI:** 10.1186/s12979-021-00230-3

**Published:** 2021-04-20

**Authors:** Christos Nikolaou, Kerstin Muehle, Stephan Schlickeiser, Alberto Sada Japp, Nadine Matzmohr, Desiree Kunkel, Marco Frentsch, Andreas Thiel

**Affiliations:** 1 Regenerative Immunology and Aging, BIH Center for Regenerative Therapies (BCRT), Charité Universitätsmedizin Berlin, Berlin, Germany; 2 Institute for Medical Immunology, Charité Universitätsmedizin Berlin, Berlin, Germany; 3 Berlin-Brandenburg School for Regenerative Therapies (BSRT), Charité Universitätsmedizin Berlin, Berlin, Germany; 4 Flow & Mass Cytometry Core Facility, Charité – Universitätsmedizin Berlin and Berlin Institute of Health (BIH), Berlin, Germany

**Keywords:** Immunoaging, Wild immunology, Adaptive immune system, Innate immune system, Bone microenvironment, Mass cytometry

## Abstract

**Background:**

Immune ageing is a result of repetitive microbial challenges along with cell intrinsic or systemic changes occurring during ageing. Mice under ‘specific-pathogen-free’ (SPF) conditions are frequently used to assess immune ageing in long-term experiments. However, physiological pathogenic challenges are reduced in SPF mice. The question arises to what extent murine experiments performed under SPF conditions are suited to analyze immune ageing in mice and serve as models for human immune ageing. Our previous comparisons of same aged mice with different microbial exposures, unambiguously identified distinct clusters of immune cells characteristic for numerous previous pathogen encounters in particular in pet shop mice.

**Results:**

We here performed single cell mass cytometry assessing splenic as secondary and bone marrow as primary lymphoid organ-derived leukocytes isolated from young versus aged SPF mice in order to delineate alterations of the murine hematopoietic system induced during ageing. We then compared immune clusters from young and aged SPF mice to pet shop mice in order to delineate alterations of the murine hematopoietic system induced by physiological pathogenic challenges and those caused by cell intrinsic or systemic changes during ageing. Notably, distinct immune signatures were similarly altered in both pet shop and aged SPF mice in comparison to young SPF mice, including increased frequencies of memory T lymphocytes, effector-cytokine producing T cells, plasma cells and mature NK cells. However, elevated frequencies of CD4^+^ T cells, total NK cells, granulocytes, pDCs, cDCs and decreased frequencies of naïve B cells were specifically identified only in pet shop mice. In aged SPF mice specifically the frequencies of splenic IgM^+^ plasma cells, CD8^+^ T cells and CD4^+^ CD25^+^ Treg were increased as compared to pet shop mice and young mice.

**Conclusions:**

Our study dissects firstly how ageing impacts both innate and adaptive immune cells in primary and secondary lymphoid organs. Secondly, it partly distinguishes murine intrinsic immune ageing alterations from those induced by physiological pathogen challenges highlighting the importance of designing mouse models for their use in preclinical research including vaccines and immunotherapies.

**Supplementary Information:**

The online version contains supplementary material available at 10.1186/s12979-021-00230-3.

## Background

Ageing of the immune system is associated with increased risk of infections as well as an increased tendency towards autoimmunity and diverse immunopathologies [1–3]. On the one hand, physiological processes such as cellular senescence, stem and progenitor cell exhaustion as well as genomic instability may represent intrinsic key factors in age-associated alterations of the immune system [4–6]. On the other hand, constant challenges with pathogens and external physical stressors induce changes in the immune system [7–9]. Murine experimental models have been widely used to assess immune ageing related alterations. However, exploring mechanisms of ageing in murine models and translating obtained results to human physiology is challenging. Laboratory mice are characterized by a diminutive lifespan compared to humans and are usually kept under non-physiological clean specific pathogen free (SPF) conditions. We and others have shown that SPF-housed laboratory animals as compared to animals living in more natural `dirty´ environments drastically differ with respect to various phenotypical and functional immune signatures [10–12]. Shielding of the immune system under SPF or germ-free conditions may enable the identification of intrinsic cellular immune alterations. We here applied multidimensional mass cytometry to delineate differences in phenotypic and functional immune signatures in primary and secondary lymphoid organs (bone marrow and spleen, respectively) of old and young SPF mice, as well as pet shop mice from our previous study as the most characteristic mouse group with distinguished immune signatures for numerous previous pathogen encounters. Our analysis identified distinct clusters of leukocytes similarly altered in old SPF mice as well as in pet shop mice. However, specific individual changes were identified in SPF-aged mice as well as in pet shop mice. This emphasizes that, while certain aspects of immune ageing and related diseases may be studied in standard experimental mouse models, common SPF housing settings may have to be adapted in order to translate results to be relevant for a physiological human setting.

## Results

### Splenic and bone marrow leukocyte composition in old versus young SPF mice

**Fig. 1:**
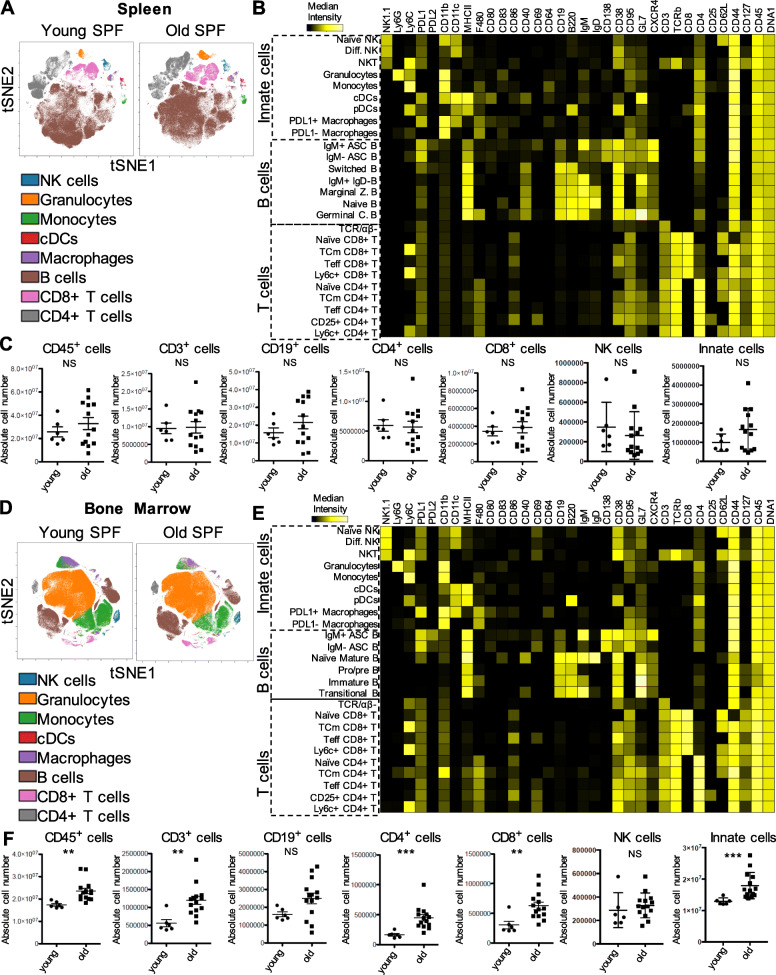
Leukocyte composition in spleen and bone marrow of young and old SPF mice. **a** and **d** Identification of differentially distributed cellular phenotypes by t-SNE in concatenated spleen and bone marrow samples respectively (Young *n* = 6 and old *n* = 13). **b** and **e** The heatmaps show median marker intensities within each population in spleen and bone marrow respectively. **c** and **f** Absolute number of selected cell populations for spleen and bone marrow (per hinge) respectively. Line indicates mean, ± SD, **P < 0.01 ***P < 0.001 Mann-Whitney test. NS indicates non-significant changes

To elucidate intrinsic cellular induced age-associated immune alterations, we first assessed changes in overall leukocytes subsets in bone marrow and spleen of young (6 months) versus old (20 months) SPF mice. Using manual gating (Supplemental Fig. 1) and unsupervised raw data clustering [13–16] of single live CD45^+^ cells, we defined established subsets such as B cells, CD4^+^, CD8^+^ T and innate cells and compared their counts and frequencies in samples from young and old SPF mice. This analysis allowed us to directly identify phenotypic differences between both SPF groups in spleen and bone marrow (Fig. 1a and d). We then analyzed each immune cluster of three major leukocyte groups: (a) CD45^+^ CD138^−^ CD3^−^ TCRβ^−^ CD19^−^ innate cells, (b) B cells and (c) T cells and identified unique populations according to cell surface markers expression for each group of mice as indicated in the heat maps for spleen and bone marrow (Fig. 1b and e). Stratifying the data from spleen and bone marrow by the relative immune cell composition, only minor differences between young and old mice could be determined (Supplemental Fig. 2). However, slightly reduced frequencies of CD4^+^ T cells and NK cells could be detected in the spleen of old SPF mice compared to the younger counterparts (Fig. 1a and Supplemental Fig. 2 left ). In contrast, the CD4^+^ T cell frequencies in the bone marrow were higher in old mice when compared to young SPF mice (Fig. 1d and Supplemental Fig. 2 right ). When comparing absolute numbers by acquisition of a defined volume of the cell suspension, counts in the spleen for CD45^+^, CD3^+^, CD4^+^ and CD8^+^ T cells as well as CD19^+^ B, NK and CD45^+^ CD138^−^ CD3^−^ TCRβ^−^ CD19^−^ innate cells were similar in both groups (Fig. 1c). In contrast, absolute counts in the bone marrow were significantly higher for CD45^+^, CD3^+^, CD4^+^ and CD8^+^ T cells as well as CD45^+^ CD138^−^ CD3^−^ TCRβ^−^ CD19^−^ innate cells in old SPF mice, while CD19^+^ B and NK cells were similar in both groups (Fig. 1f). Altogether, CD45^+^ and major immune subset absolute counts between young and old SPF mice were similar in the spleen, in contrast to the bone marrow where increased absolute cell numbers indicate the accumulation of immune cells in aged bone marrow environment. Based on their frequencies, CD4^+^ T cells were less abundant in the spleen in contrast to the bone marrow of old SPF mice compared to the young SPF mice.

### Differentiated innate cells in spleen in old versus young SPF mice

**Fig. 2:**
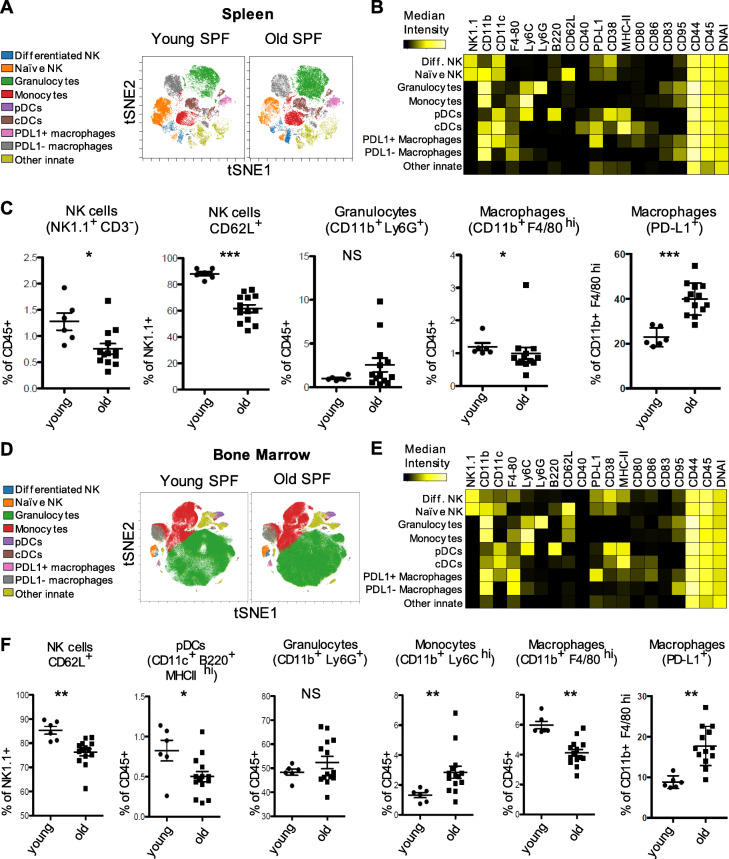
Age related changes in the innate cell composition in spleen and bone marrow of SPF mice. **a** and **d** Identification of differentially distributed cellular phenotypes by t-SNE in concatenated spleen and bone marrow samples respectively (Young *n* = 6 and old *n* = 13). **b** and **e** The heatmaps show median marker intensities within each population in spleen and bone marrow respectively. **c** and **f** Selected innate populations were gated according to established lineage markers (Supplemental Fig. 1 for gating strategy). Line indicates mean, ± SD, *P < 0.05, **P < 0.01 ***P < 0.001 Mann-Whitney test. NS indicates non-significant changes

In order to study age-related changes among innate subsets we analyzed CD45^+^ CD138^−^ CD3^−^ TCRβ^−^ CD19^−^ innate cell populations such as naïve and mature NK cells, granulocytes, monocytes, macrophages, plasmacytoid dendritic cells (pDCs) and conventional dendritic cells (cDCs), as well as their activation associated marker signatures (Fig. 2a, b and Supplemental Fig. 1). We observed significantly lower frequencies of splenic NK cells and specifically a reduction of naive CD62L^+^ NK cells among NK1.1^+^ cells as well as a minor reduction of macrophages in old animals (Fig. 2c). Furthermore, differentiated classical activated macrophages characterized by F4/80^+^, PD-L1^+^ expression, as described by Loke and coworkers [17] were more frequent in old SPF mice compared to the young SPF group (Fig. 2c). In contrast, we found no changes in splenic frequencies of granulocytes (Fig. 2c), pDCs, cDCs and monocytes among young and old SPF mice (Supplemental Fig. 4A). Altogether, distinct innate subsets such as NK cells and macrophages acquired differentiated phenotypes in the spleen of old mice.

### Differentiated innate cells in bone marrow in old versus young SPF mice

To investigate whether myelopoiesis is affected during ageing, we also analyzed the composition of CD45^+^ CD138^−^ CD3^−^ TCRβ^−^ CD19^−^ innate cells in the bone marrow as a primary lymphoid organ (Fig. 2d and e). We found no changes in frequencies of granulocytes (Fig. 2f), cDCs and NK cells (Supplemental Fig. 3A) between old and young SPF mice. However, lower frequencies of naïve CD62L^+^ NK cells among NK1.1^+^ cells and a decrease of macrophages in old SPF mice were demonstrated. In contrast to the results from spleen, we identified significant less pDCs but increased frequencies of monocytes (Fig. 2f). Taken together these data indicate that the hematopoietic generation of granulocytes and cDCs in the bone marrow is maintained over the lifespan of SPF laboratory mice, whereas hematopoiesis and differentiation towards monocytes, differentiated PD-L1^+^ macrophages and CD62L^−^ NK cells (Fig. 2f) are increased with age.

### Antibody secreting cells and ‘switched’ B cells in spleen in old versus young SPF mice

**Fig. 3:**
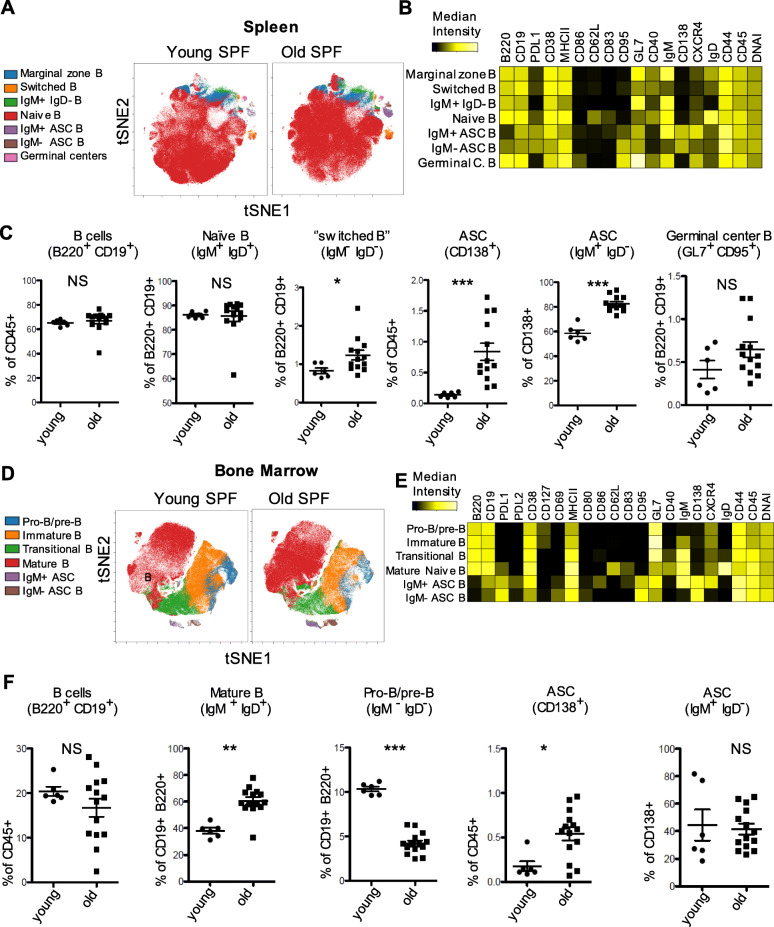
Age related changes in the development of B cells in spleen and bone marrow of SPF mice. **a** and **d** Identification of differentially distributed cellular phenotypes by t-SNE in concatenated spleen and bone marrow samples respectively (Young *n* = 6 and old *n* = 13). **b** and **e** The heatmaps show median marker intensities within each population in spleen and bone marrow respectively. **c** and **f** Selected populations of B cells were gated according to established lineage markers (Supplemental Fig. 1 and 3B for gating strategy). Line indicates mean, ± SD, *P < 0.05, **P < 0.01 ***P < 0.001 Mann-Whitney test. NS indicates non-significant changes

Previous studies have shown that B cell proliferation and differentiation from lymphoid progenitor to mature B cells is impaired in aged mice compared to young mice [18, 19]. In order to evaluate age related effects on mature subsets of B cells, we assessed markers such as IgM, IgD, MHC-II, CD40, CD62L, CD80, PD-L1, CD38, GL7, CD95, CXCR4, CD138 and CD127 on B cells (Fig. 3a, b and Supplemental Fig. 1). We found similar frequencies of total (CD19^+^ B220^+^) and naïve (IgM^+^ IgD^+^) B cells in both groups (Fig. 3c), while slightly lower frequencies of marginal zone (IgM^+^ IgD^low^) B cells were observed in the older group (Supplemental Fig. 4B). Higher frequencies of antigen-experienced IgD^−^ IgM^−^ “switched” B cells were found in old SPF mice, indicating that even under long term SPF conditions a substantial pool of differentiated B cells is formed (Fig. 3c). Notably, old mice showed accumulation of total CD138^+^ antibody secreting cells (ASCs) and specifically IgM^+^ IgD^−^ ASCs (Fig. 3c). Germinal center (GC, GL7^+^ CD95^+^) B cells were found equally low in both groups demonstrating that no acute immune responses were present at the time of analysis (Fig. 3c). In summary, these results demonstrate a significant accumulation of antigen-experienced B cells and particularly IgM^+^ IgD^−^ ASCs in the spleen of old mice housed under clean SPF conditions.

### Decreased pro-B/pre-B cells but increased mature naive B cells in bone marrow in old versus young SPF mice

The literature is contradictory regarding B cell subset frequencies and numbers in murine bone marrow [18–22]. We wanted to evaluate whether differences in the B cell composition are a result of age-related changes in B cell development at their place of origin (Fig. 3d-f). We observed similar frequencies of total (CD19^+^ B220^+^) B cells but a significant decrease of frequencies in pro-B/pre-B cell (IgM^−^, IgD^−^, CD127^+^) compartment (Fig. 3f), but also in immature (IgM^low^, IgD^−^, CD127^+^) and transitional (IgM^high^, IgD^+−^, CD127^−^) B cells in aged mice (Supplemental Fig. 3B). In contrast, frequencies of mature naïve (IgM^+^, IgD^+^, CD127^−^) B cells were increased in aged mice (Fig. 3f). Some immune cells such as distinct subsets of memory B- and T-cells as well as plasma cells reside in the bone marrow [23]. In contrast to spleen, we did not observe any significant difference in IgM^+^ IgD^−^ ASCs between the two groups (Fig. 3f). However, the frequencies of total CD138^+^ ASCs in the bone marrow were higher in old compared to young mice (Fig. 3f). Taken together, despite the reduction in pro-B/pre-B cell compartment, the frequency of mature naïve B cells in bone marrow was higher than expected, while IgM^+^ IgD^−^ ASCs were more abundant in the spleen instead of the bone marrow of old SPF mice.

### Age-related increase of T effector and central memory subsets

**Fig. 4:**
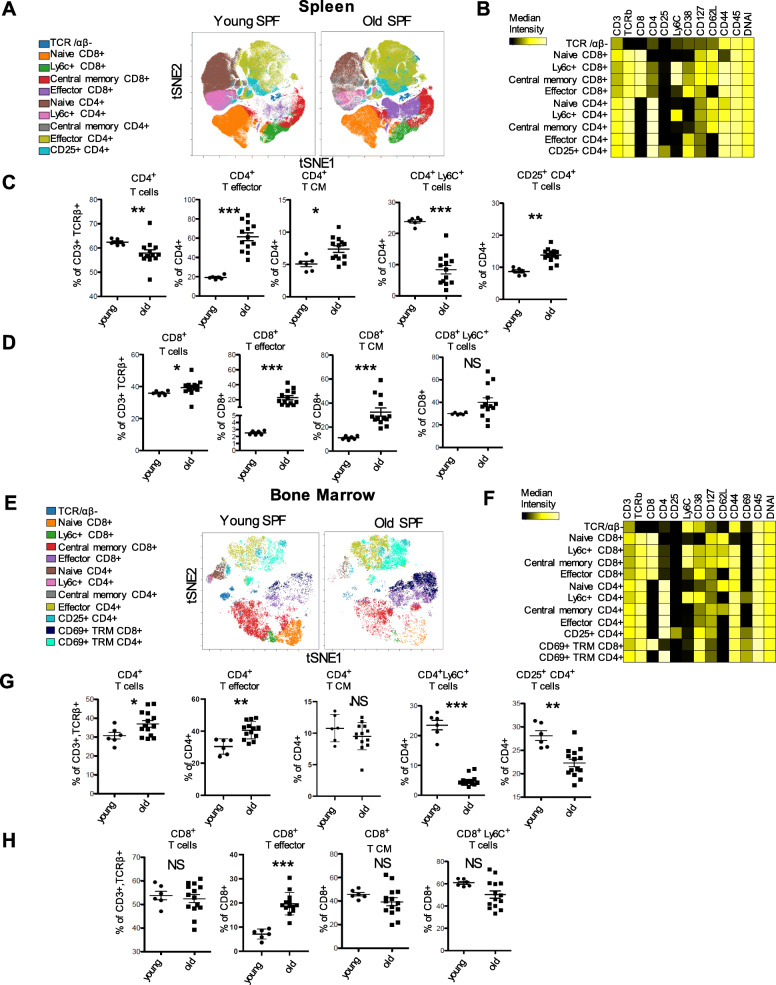
Age related changes in T cell subsets in spleen and bone marrow of SPF mice. **a** and **e** Identification of differentially distributed cellular phenotypes by t-SNE in concatenated spleen and bone marrow samples respectively (Young *n* = 6 and old *n* = 13). **b** and **f** The heatmaps show median marker intensities within each population in spleen and bone marrow respectively. **c** and **g** Selected populations of CD4+ T cells. **d** and **h** Selected populations of CD8+ T cells. Populations of T cells were gated according to established lineage markers (Supplemental Fig. 1 and 3 for gating strategy). Line indicates mean, ± SD, *P < 0.05, **P < 0.01 ***P < 0.001 Mann-Whitney test. NS indicates non-significant changes

Involution of the thymus and the associated diminished output of naïve T cells represents one of the most recognized changes in the immune system with age [24–26]. We delineated CD4^+^ and CD8^+^ T cells in naïve (Tnaive, CD62L^+^ CD44^−^), effector (Teff, CD62L^−^ CD44^+^), and central memory (TCM, CD62L^+^ CD44^+^) T cells according to expression of CD44 and CD62L (Fig. 4a, b and Supplemental Fig. 1). Old SPF mice revealed the lowest frequencies of CD4^+^ T cells in the spleen in comparison to young SPF mice. Specifically, higher frequencies of effector CD4^+^ T cells and central memory CD4^+^ T cells were observed in the spleen of old mice (Fig. 4a and c). In addition, Ly6C^+^ CD4^+^ T cells were decreased in old mice, indicating an increase in Th1 memory precursor cells with higher survival and proliferative capacities [27]. High CD25 expression on murine CD4^+^ T cells predominantly characterizes regulatory T cells, which were increased in the old SPF mice in comparison to the young SPF animals (Fig. 4c).

In contrast to CD4^+^ T cells, old SPF mice exhibited significantly higher frequencies of total CD8^+^ T cells (Fig. 4d). Analogous to CD4^+^ T cells, old mice displayed more effector and central memory cells in their CD8^+^ T cell compartment (Fig. 4a and d). Since Ly6C is known to be expressed on subsets of memory CD8^+^ T cells [28, 29], we expected higher frequencies in the old group, which exhibited the largest CD8^+^ effector and central memory T cell compartments. Surprisingly and in contrast to CD4^+^ T cells, we observed no significant differences between old and young Ly6C^+^ CD8^+^ T cells in the spleen (Fig. 4d). In summary, these results indicate an overall increase of “immunologically experienced” CD8^+^ and CD4^+^ T lymphocytes in the spleen of old SPF mice compared to young SPF mice.

### Effector, central memory and CD69^+^ tissue resident memory bone marrow T subsets in old versus young SPF mice

Next we wanted to study age-related changes of T cells in the bone marrow representing a site for migration or selective retention of subsets of distinct memory T cell subsets [30–32]. For that purpose, T cell subsets were assessed in the bone marrow of young and old SPF mice (Fig. 4e and f). Effector CD4^+^ T cells were highly increased in the bone marrow of old SPF mice (Fig. 4e and g). Notably, frequencies of central memory CD4^+^ T cells remained unaffected in old SPF mice in contrast to the spleen, while Ly6C^+^ CD4^+^ T cells were reduced as demonstrated before in the spleen (Fig. 4g). In contrast to the spleen, CD25^+^ CD4^+^ T cells were reduced in the bone marrow of old SPF mice in comparison to their young SPF counterparts (Fig. 4g). We detected significantly elevated effector CD8^+^ T cells in bone marrow of old mice (Fig. 4e and h). Central memory and Ly6C^+^ CD8^+^ T cells showed no significant changes between the groups (Fig. 4h). Bone marrow tissue resident (TRM) CD4^+^ and CD8^+^ T cells were identified based on residency marker CD69 expression [31, 33] (Supplemental Fig. 3D). Both CD4^+^ and CD8^+^ CD69^+^ CD62L^−^ CD44^+^ tissue resident memory T cells (TRM) were increased in the old group (Fig. 4e-f and Supplemental Fig. 3D-E). In summary, these results show a decrease of CD25^+^ CD4^+^ T cells and support a gradual increase of differentiated CD4^+^ Teff and TRM as well as CD8^+^ Teff and TRM lymphocytes in the bone marrow with increasing age.

### Cytokine expression of CD4^+^ and CD8^+^ T cells in spleen of old versus young SPF mice

**Fig. 5:**
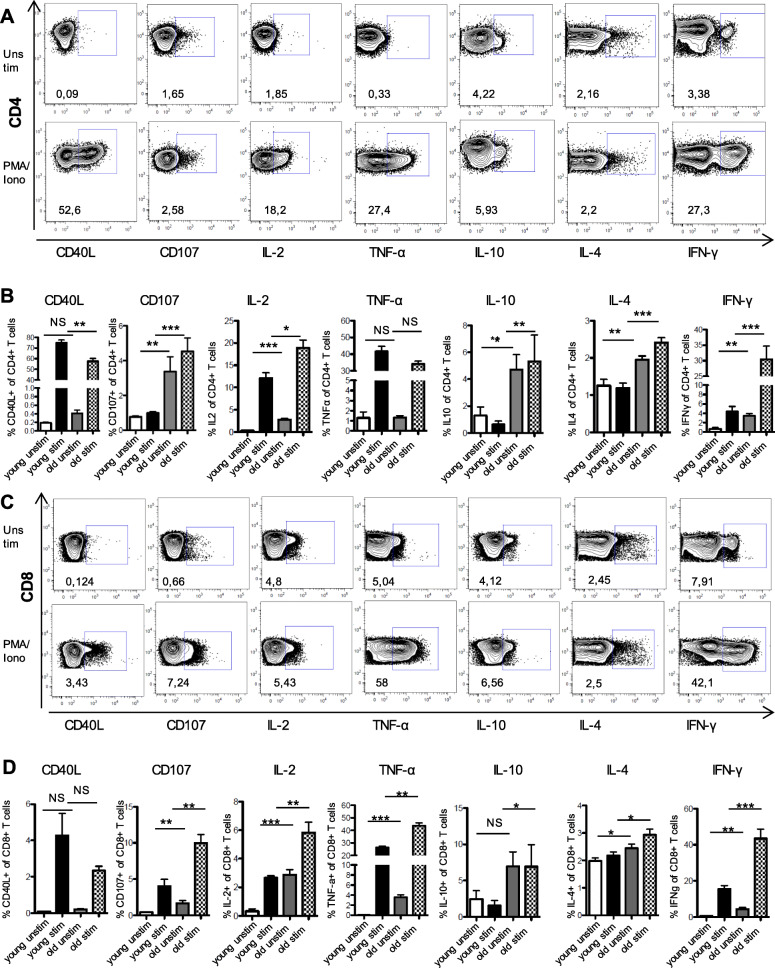
Cytokine secretion profile of CD4+ and CD8+ T cells is altered according to age in spleen of SPF mice. **a** and **c** Contour plots showing the cytokine gates for CD4+ (**a**) and CD8+ T cells (**c**) in unstimulated and 5 hours, phorbol-myristate-acetate and ionomycin stimulated samples from a representative old SPF mouse spleen sample. **b** and **d** Summary of the frequencies of cytokine-positive cells in CD4+ (**b**) and CD8+ T cells (**d**) *P < 0.05, **P < 0.01, ***P < 0.001 Mann-Whitney test. NS indicates non-significant changes. (Young *n* = 6 and old *n* = 13)

Compared to young SPF mice, old SPF mice showed higher frequencies of antigen-experienced T cells in the spleen. To obtain more insights into the functional diversity of these T cells, we assessed the production of effector cytokines (IFN-γ, IL-2, TNF-α, IL-4 & IL-10) as well as CD40L (CD154) and lysosomal-associated membrane protein 1 (CD107a) expression after 5 h polyclonal stimulation (with phorbol-myristate-acetate and ionomycin) (Fig. 5a and c). Old SPF mice contained significantly higher frequencies of IL2-, IL10-, IL4- and IFN-γ-producing CD4^+^ T cells as compared to young SPF mice (Fig. 5b). In contrast, TNF-α-producing CD4^+^ T cells remained similar in both groups (Fig. 5b). Taken together, these results are in line with the phenotypic analysis described above demonstrating an increase in effector and central memory CD4^+^ T cells in the spleen of old SPF mice (Fig. 4a-c). The reduction in frequency of CD40L^+^ CD4^+^ T cells was accompanied by an increase of cytotoxic CD107^+^ CD4^+^ T cells in the aged SPF mice (Fig. 5b). In a similar manner, splenic effector cytokine-producing CD8^+^ Τ cells were significantly increased in old mice (Fig. 5d). Interestingly, CD40L^+^ helper-type CD8^+^ T cells remained similar in both groups, while cytotoxic CD107^+^ CD8^+^ T cells were increased in aged mice (Fig. 5c-d). Using Boolean gating, we detected higher frequencies of poly-functional CD4^+^ as well as CD8^+^ T cells in old mice (Supplemental Fig. 5). In conclusion, changes in cytokine profiles of CD4^+^ and CD8^+^ T cells in the spleen correlate with the decline of naïve T cells and show an increase in specific effector T cell subsets in old SPF mice.

### Immune cell signatures in the spleen of pet shop versus old SPF mice

**Fig. 6:**
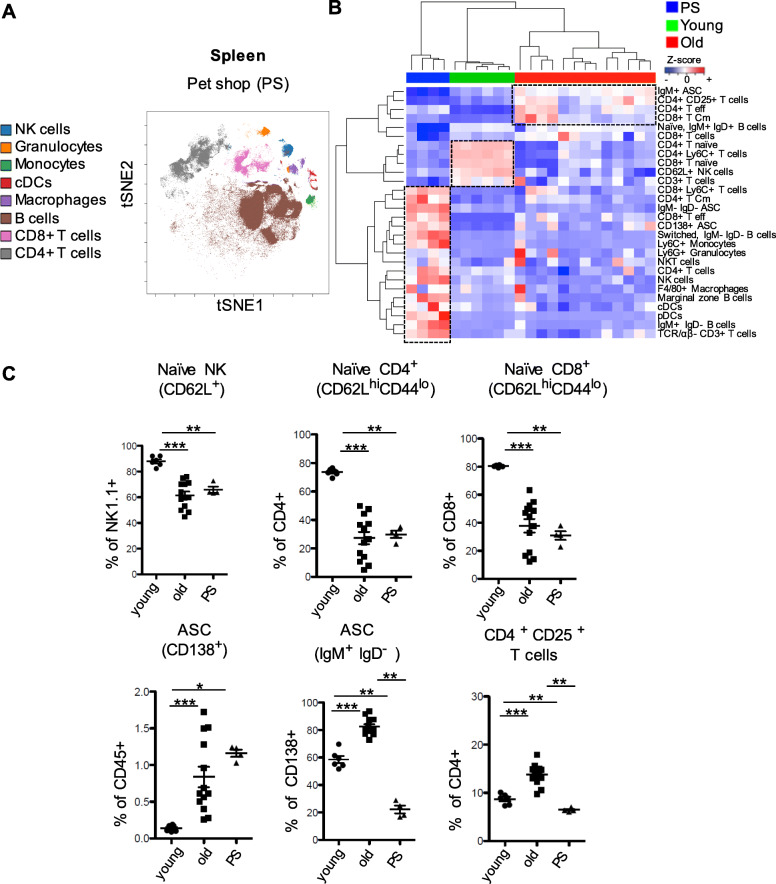
Young, old SPF and pet shop mice differ in their overall CD45+ splenic leukocyte composition. **a** Identification of differentially distributed cellular phenotypes by t-SNE in concatenated pet shop spleen samples (*n* = 4). **b** The heatmap shows z-scores of population frequencies of major immune population identified in spleen between all three mouse groups. Heat map coloring has been scaled per row, higher than mean (red) and lower than mean (blue). Each cluster of correlating populations (dashed boxes), features immune signatures that distinguish the three experimental groups. **c** Selected naïve and differentiated immune populations in the spleen characteristic among all three mouse groups. Line indicates mean, ± SD, *P < 0.05, **P < 0.01 ***P < 0.001 Mann-Whitney test

Our last comprehensive assessment of the murine immune system by comparing mice of same age in our murine housing facilities with potentially different microbial exposures, namely SPF laboratory mice, SPF mice housed for 2 months under non-SPF conditions, mice bred in quarantine, and pet shop mice unambiguously identified distinct clusters of myeloid and lymphoid cells that are characteristic for numerous previous pathogen encounters in particular in pet shop mice [10]. We next compared data sets on the composition of splenic cells isolated from pet shop mice from our recent study [10] as the most characteristic mouse group with distinguished immune signatures for numerous previous pathogen encounters to the data sets obtained here from old and young SPF mice (Fig. 6a-c and Supplemental Fig. 4). Due to perpetual pathogen challenges pet shop mice should at least partially serve as a model for physiological age-associated immune changes. On the one hand, pet shop mice had the highest frequencies of splenic innate subsets such as total NK cells, granulocytes, pDCs, cDCs and monocytes compared to both young and old SPF mice (Fig. 6b and Supplemental Fig. 4Α). Moreover, frequencies of marginal zone (MZ), IgM^−^ IgD^−^ “switched” B cells (Fig. 6b and Supplemental Fig. 4B) and total CD138^+^ ASCs (Fig. 6b and c) were the highest in the pet shop mice. Significant higher frequencies of total CD4^+^ T cells, CD4^+^ central memory, CD8^+^ effector and CD8^+^ Ly6C^+^ T cells were also observed in the pet shop mice among all three groups (Fig. 6b and Supplemental Fig. 4C). On the other hand, pet shop mice displayed reduced frequencies of naïve CD62L^+^ NK cells, naïve CD4^+^ and naïve CD8^+^ T cells equally to the old SPF mice (Fig. 6b and c). Pet shop mice, displayed most reduced frequencies of CD138^+^ IgM^+^ IgD^−^ ASCs as well as CD4^+^ CD25^+^ T cells (Fig. 6b and c), naïve IgM^+^ IgD^+^ B cells, total CD3^+^ T cells and total CD8^+^ T cells when compared to both SPF groups (Fig. 6b and Supplemental Fig. 4D). In summary, naïve subsets such as naïve CD62L^+^ NK, CD4^+^ and CD8^+^ T cells were similarly decreased in old SPF and pet shop mice while naïve B cells were reduced only in the pet shop group. Interestingly, even though both old SPF mice and pet shop mice showed increased experienced B cell subsets, old SPF mice accumulated more CD138^+^ IgM^+^ IgD^−^ ASC B cells (Fig. 6c), which may develop even in the absence of germinal centers and can be retained within the spleen [34]. Thus, old mice kept under SPF conditions have a mature immune system along with some typical immune ageing signatures like decline of frequencies of total CD4^+^ T cells as well as increase of total and central memory CD8^+^ T cells as well as Treg frequencies in comparison to the immune system of pet shop mice (Fig. 6b-c and Supplemental Fig. 4C-E). In contrast, pet shop mice facing perpetual physiological microbial challenges demonstrate a variety of immune cells such as innate subsets like granulocytes, NK cells, pDCs, cDCs and monocytes as well as CD138^+^ IgM^−^ IgD^−^ ASC B cells.

## Discussion

Ageing of the immune system is a multifactorial process, driven by intrinsic and extrinsic mechanisms such as cellular senescence, stem cell exhaustion, genomic instability but also physiological acute and chronic pathogen challenges. In parallel, accumulating evidence suggests that important physiological parameters such as microbiome configuration, diet, sterile derivation and husbandry practices can modulate the mammalian innate and adaptive immune system by regulating a delicate balance of pro- and anti-inflammatory responses [10, 11, 35–38]. We here examined individual immune signatures of healthy young and old SPF mice and compared these to the immune signatures of healthy pet shop mice. One of our aims was to elucidate in parallel innate and adaptive immune differences between young and old SPF animals not only in the spleen but also in the bone marrow thereby covering secondary and primary lymphoid organs respectively. High-dimensional single cell mass cytometry permitted to overcome experimental limitations by enabling the monitoring of multiple cell subsets or correlates within our study carrying the potential to understand the immune networking at a certain time point at a deeper level. Additionally, we aimed to dissect possible intrinsic and extrinsic consequences onto the immune maturation and ageing. Shielded partially from pathogens and commensals old SPF mice may partly be suitable to assess age-associated changes in the immune system that are concerted by immune cell or system intrinsic mechanisms. While germ-free mice would fit best to assess such intrinsic immune ageing alterations, it has been demonstrated already more than 50 years ago that they exhibit longer life-spans as compared to control mice [39]. More recently it has been demonstrated that aged gut microbiota isolated from mice bred under conventional conditions can contribute to systemic inflammageing [40]. We could delineate distinctly altered immune signatures in adult pet shop mice from alterations in old SPF mice most likely resulting from cell intrinsic or systemic changes during ageing. All of our mice weighted over 25 g and were considered adults. Even though we could not precisely identify the age of pet shop mice we considered them as adults, in the range of 4–8 months old, based on some of their physical attributes such as weight, fur and dental anatomy [41–44]. In particular, we analyzed and categorized three major immune clusters, 1) innate subsets, 2) B cell subsets and 3) T cell subsets.

Older SPF mice exhibited alterations such as less naïve CD62L^+^ NK cells and more differentiated CD62L^−^ NK cells and PD-L1^+^ macrophages both in the spleen and bone marrow in contrast to young SPF mice. CD62L^−^ differentiated NK cells have been already described in mice and humans [45]. Likely due to endogenous accumulation of natural inflammatory events such as oxidative metabolism which leads to increased reactive oxygen species (ROS) and consequently oxidative stress and cellular debris [46] in combination with few microbial encounters present in our SPF facility, more NK cells and classically activated macrophages acquired such a differentiated phenotype in old SPF mice. Such differentiated innate CD62L^−^ NK cells may resign in the bone marrow. We hypothesized that similar to T cells, expression of residency marker CD69 known for tissue retention could also be a mechanism that could retain NK cells in the bone marrow. Interestingly, we have not found a significant difference among CD69^+^ CD62L^−^ NK cells in the bone marrow of young vs old SPF mice (Supplementary Fig. 3A). On the one hand, CD69^+^ NK cells have been shown to increase in the bone marrow of mice and peaked 72 h MHV-3 post-infection [47] which would support our observations that no acute immune responses were present at the time of analysis. Even though CD69 expression has been reported in NK bone marrow resident cells the ageing murine tissue resident NK cells can present specific characteristics that involve additional/alternative resident mechanisms for example expression of chemokine receptors CXCR6, CCR5, CCL3 and CXCL12 [48–50].

Overall in the innate subsets, the comparison of the spleen data among all three mouse groups revealed that the pet shop mice had the highest abundancy of granulocytes, pDCs, cDCs and monocytes, indicating better equipped first lines of defense adapted against a multitude of potential pathogens, including viruses, bacteria and endoparasites.

We revealed extensive and profound changes not only in innate but also adaptive immune cell lineages specifically in distinct subsets of effector/memory B cells. In the bone marrow, altered B lymphopoiesis such as distinctly diminished frequencies of pro-B/pre-B cell cells in old mice is likely resulting from the loss of B-lineage precursors, which is in line with the results from Miller and Allmann et al. [19]. Despite this, numbers and frequencies of mature naïve IgM^+^ IgD^+^ B cells in bone marrow were higher in old compared to young SPF mice. In contrast, numbers and frequencies of naïve IgM^+^ IgD^+^ B cells in the spleen were similar between young and old SPF mice. This is in line with other studies reporting a decline in pre-B cells in aged mice without a decrease in the number of naïve IgM^+^ IgD^+^ B cells [18, 22, 51]. The increase of mature naïve IgM^+^ IgD^+^ B cells in old SPF mice can be justified by reduction of positive and negative selection mechanisms that normally regulate the pre-B-to-B cell transition thus allowing more B cells to escape selection and tolerance promoting possibly age-associated autoimmune diseases [21, 52]. Notably, old SPF in contrast to young SPF mice exhibited more ASCs both in spleen and bone marrow, whereas physiological low frequencies of germinal center B cells were equal in spleen for both SPF mouse groups. Interestingly, old SPF in contrast to pet shop animals showed accumulation of single IgM^+^ ASCs in the spleen. Bohannon and coworkers showed that IgM^+^ ASCs are primarily retained within the spleen and can develop even in the absence of germinal centers [34]. Consequently, the increase of IgM^+^ ASCs in old SPF mice can be explained by considering a long life-time under lack of firstly corresponding T cells and secondly direct microbial stimuli, both crucial for a broad variety of ASCs and isotype switch. This effect strongly suggests that even though total ASCs were elevated in both old SPF and pet shop mice, the pet shop mice had developed a broad variety of antigen experienced B cells, demonstrated by low frequencies of naïve B cells and high frequencies of CD138^+^ IgD^−^ IgM^−^ ASCs instead of CD138^+^ IgD^−^ IgM^+^ ASCs developed in old SPF mice, representing most likely the pet shop’s mice natural exposure to a plethora of microbes.

From the T lymphocytes’ perspective, aged as compared to young SPF mice demonstrated strong signs of naïve T cell decline. While bone marrow is not a major primary entry site for pathogens and antigenic challenges, it represents a location to preserve or stabilize at least a fraction of long-term cellular memory by shielding it from potential bystander activation that is frequently present in secondary lymphoid organs. Additionally, immune cell including T cell interactions with hematopoietic precursor populations have been shown to maintain and regulate hematopoiesis [53–56]. Absolute cell numbers of total CD4^+^ and frequencies of effector as well as CD44^+^ CD69-expressing TRM CD4^+^ and CD8^+^ T cells were increased in aged as compared to young SPF mice. In comparison, frequencies of central memory CD4^+^ and CD8^+^ T cells were similar in both young and old mice while CD4^+^ CD25^+^ Tregs were decreased in the old group. Notably, we found no changes between young and old SPF mice in frequencies neither of TCRβ+ Natural killer T (NKT) cells nor TCRβ^+^ CD62L^+^ NKT cells. In contrast, frequencies of TCRβ^−^ NKT cells and TCRβ^−^ CD62L^+^ NKT cells were reduced in the old group in comparison to young SPF (Supplemental Fig. 3C) indicating different compositions of NKT subsets recognizing self and foreign lipids in aged bone marrow environment.

Overall, in the aged compared to young bone marrow we detected on the one hand 1) lower frequencies of pDCs, higher frequencies of monocytes and PDL1^+^ macrophages. On the other hand, in the aged compared to young bone marrow we detected 2) lower frequencies of Tregs, higher frequencies of T effector and TRM while TCM cells and IgM^+^ ASCs remained unaltered. Characterization of single cells acquired at a certain time point with multi-parametric methodologies might potentially lead to misinterpretation regarding immune subsets involved in protection or misestimating of certain parameters in assessment of immune responses. Nevertheless, analysis of such age-related changes between innate and adaptive cells in the BM microenvironment could allow us to better understand possible networks and spatial competitions between memory subsets but also regulations of hematopoiesis in murine and human aged bone marrow niches; for example, under marrow adipose tissue accumulation, marrow remodeling and bone loss which occur during aging [57–59].

In the spleen similar to pet shop mice, old SPF mice revealed a higher frequency of effector and central memory CD4^+^ and CD8^+^ T cells, which exhibited increased frequencies of effector-cytokine producing T cells. CD4^+^ CD25^+^ Treg cells were more abundant in the spleen of old SPF mice in comparison to young SPF and pet shop mice. Notably, the decline of naïve CD8^+^ and CD4^+^ T cells and concomitant increase of Tregs represent some of the most distinctive age associated alterations in cell signatures in both mouse and human [24, 25, 60]. Interestingly, recent results from scRNA-seq have revealed unique complex gene expression signature of CD4^+^ T cells in aged mice most notably, the aTregs, exhausted, and cytotoxic CD4^+^ subsets [61]. The CD4^+^ cytotoxic subset appeared to be dominantly regulated by transcription factors associated with cytotoxicity and T helper 1 (Th1) polarization, including *Eomes, Runx2, Runx3, and Tbx21* regulons which are also required for the cytotoxic CD8 lineage [61, 62]. This result is in line with our effector-cytokine producing CD4^+^ T cells data where we observed a significant increase of cytotoxic CD107^+^ CD4^+^ T cells and polyfunctional CD107^+^ IFN-γ^+^ CD4^+^ T cells lacking typical helper CD40L, IL2 and IL4 expression (Supplemental Fig. 5). Cytotoxic CD4^+^ cells were previously identified and therapeutically used in murine models of cancer and colitis [63, 64] and in healthy humans and human viral infection [62, 65]. However, they have not been comprehensively linked to aging nor CD107^+^ (LAMP-1) has been directly assessed in that context and while the antigen specificity, differentiation pathways, function, and accumulation of cytotoxic CD4^+^ cells in aging are yet to be revealed, they may mark a stage in aging with a robust immune failure and chronic inflammation or in contrast an adaptation to the late stage of aging in supercentenarians humans [66].

Even though analyzing multiple organs was not in the scope of our study, age-related changes occur in the cellular composition of both lymphoid and non-lymphoid organs. As expected, our results are in line with published scRNA-seq results from the spleen where the proportion of T cells decreased with age while the relative amount of plasma cells increased supported by upregulation of *Cd79a* and *Jchain* and the downregulation of *Cd3d* [67]. Analysis of clonal relationships between B cells and T cells throughout the organism revealed the number of B cells and T cells belonging to a clonotype doubled at 18 months (20–23%) when compared to young mice indicating an increase in clonality of the B and T cell repertoire at later ages [67] which is what we hypothesize and expect in our aged SPF mice in comparison to young or pet shop mice. These changes in clonality for both B cell and T cell repertoires between young and old mice in parallel with increased innate first line of defenses observed in the pet shop mice are noteworthy because they suggest how the immune system, for example of old mice, is less likely to respond to some pathogens. In parallel to T cell and B cell modifications in the spleen the researchers observed alteration in tissue composition of kidney, liver, skin with age in that mice as well as decreased mesenchymal compartments in the bladder with increasing age while the urothelial compartment increased and was infiltrated by a plethora of leukocytes known as age-related urothelial changes. It would be of interest to investigate in the future if our holistic detected aging changes in lymphoid organs such as the bone marrow and spleen correlate to systemic organ alterations and unravel in detail not only the composition but also the mechanisms of the immune system onto age-related organ modifications.

Although SPF mice are an invaluable platform for immunological studies, further research is needed towards establishing murine experimental set-ups that allow more closely emulation of human physiology. One key point is that the immune system of commonly used SPF mice maintains a relatively naïve state throughout the animal’s entire life. Accordingly, several immune signatures in SPF mice have similarities to those of neonatal humans [11]. While establishing the use of feral or pet shop mice in experimental settings could be challenging to implement, the use of conventional SPF mice challenged with a defined set of physiological infections, may be a suitable compromise in future experimental assessment. Being exposed to “natural” pathogens can induce normalized immune systems, suitable to study immune ageing and diseases similar as in humans, improving immune responses to infections and most notably even accurately phenocopy patient outcomes in clinical trials [11, 68, 69].

Varying degrees of immune experience in mice can be now controlled through diet, sterile derivation, husbandry practices, and sourcing genetically outbred pet store and feral mice from environments outside of biocontainment. We here show how the age of mice is also a parameter which should be taken into consideration in order to normalize, only to some extent, the immune system. We here highlight specific immune signatures imprinted by accumulating inflammatory events firstly under ageing SPF conditions, such as high abundancy of differentiated T, B and NK cells and notably Tregs and IgM^+^ ASCs. Secondly, distinct alterations are demonstrated under repetitive natural environmental microbial driven conditions, such as high abundancy of differentiated T, B and NK cells and notably ‘switched’ IgD^−^ IgM^−^ B cells, granulocytes, pDCs, cDCs and monocytes, represented in the pet shop group (Fig. 6). To avoid contamination of inbred lab mouse colonies, we were not able to perform pathogen challenges comparing different groups of SPF with “wild” mice in our facilities. A solution for that would be to perform those experiments under biosafety 3 conditions. Due to this restriction, we assessed solely phenotypic characteristics and short-term functional properties. We cannot exclude that different genetic backgrounds in pet shop mice represent an important factor influencing the differences observed, however, this seems very unlikely considering the fact that pet shop mice are infected by a multitude of pathogens, including viruses, bacteria, and endoparasites [70] .In line with that, our previous experiments comparing three murine groups with the same age sharing the exact same CD45.1, C57BL/6 J genetic background and age (a) SPF laboratory mice, (b) SPF mice housed for 2 months under non-SPF conditions and (c) mice bred in quarantine to (d) pet shop mice, revealed phenotypic and functional differences regarding the “maturity” of their immune system including absolute numbers and frequencies of immune subsets which associated strongly with the degree of pathogenic priming [10]. Particularly, analogous results on our CD45.1, C57BL/6 J quarantine mice indicate that such immune differences are mostly due to the absence of pathogen infection in SPF animals sharing exactly the same genetic background [10]. Nonetheless, due to our staining strategy of surface antigens for mass cytometry using selected monoclonal anti-bodies we can exclude specific genetic backgrounds for the pet shop group. In particular, the monoclonal ab NK1.1 (clone: PK136) used for the phenotyping of NK cells in our surface mass cytometry panel reacts only with mouse surface antigen NK1.1 in mouse strains such as C57BL/6 J, FVB/N and NZB but not AKR, BALB/c, CBA/J, C3H, DBA/1, DBA/2, NOD, SJL, and 129 murine strains which do not express the NK1.1 antigen and instead the clone DX5 should be used [71–74]. Additionally, both pet shop and quarantine mice contained significantly higher frequencies of IFNγ-producing CD8^+^ and CD4^+^ T cells similarly to our old SPF mice analyzed here as compared to young SPF and non-SPF mice. The percentage of CD4^+^ T cells producing the immunosuppressive cytokine IL-10 was increased in pet shop and quarantine mice, suggesting that immunoregulation by IL-10-producing cells is an essential part of effector T cell immunity against natural pathogens, possibly reducing immunopathology. In addition the frequencies of IL-17A^+^ CD8^+^ and CD4^+^ T cells were drastically increased in pet shop mice as compared to the other mouse groups from our previous study [10]. In line with these results, independent experiments revealed the secretion of IL-17A and IL-13 was limited to CD40L^+^, but not CD40L^−^ CD8^+^ memory T cells in immune competent pet shop mice from our most recent study [62], while such cells were barely detectable in SPF mice. Considering the strong association between the intestinal microbiota and the priming of IL-17A-producing cells [75], it is reasonable to attribute this increase to differences in the normal microbiota between pet shop mice and lab-housed mice [10].

Differential effects of age on hematopoietic progenitor cell functions, circulating and splenic leukocyte populations have been long known between strains such as C57BL/6 J, BALB/c and D2 mice [76–78]. Numerous reports suggest a preferential bias for the C57BL/6 J mouse strain to develop Th1-type response, whereas for example the BALB/c strain is biased towards a Th2-type cytokine polarization to some infectious agents [76, 79–83]. Additionally, TLR signaling and cytokine production in aged monocytes and macrophages has also been shown to be different between the BALB/c and C57BL/6 J strains [84–86]. Nevertheless, similar approaches using young substrain C57BL/6 N (in contrast to our case C57BL/6 J) and unidentified background pet shop mice in cohousing experiments showed that microbial exposure enhances immunity to pathogens recognized by TLR2 but increases susceptibility to cytokine storm through TLR4 sensitization [87]. Signatures from the blood of these mice showed similar relations and fluctuations to our young SPF mice and pet shop in particular elevated frequencies of CD4+ T cells, monocytes, Ly6G+ granulocytes and decreased frequencies of B cells in the pet shop group supporting that some major immune cell signatures which we described could be relevant to substrains and even to unidentified genetical backgrounds. Last but not least, we cannot exclude that without comparing aged pet shop mice or/and old SPF mice challenged with a controlled set of pathogens to young and old SPF mice we cannot clearly decipher aging “SPF edited” from pathogen driven immune signatures. More multidimensional immuno-profiling studies are clearly needed including bone marrow but also non lymphoid organ data in different ages of pet shop and other commonly used murine strains physiologically challenged with define microbes during their lifetime to better define and understand T and B cell memory subsets but also systemic immune alterations during ageing from physiological pathogen challenges.

## Conclusions

The data presented here demonstrate a) how ageing impacts both innate and adaptive immune cells not only in spleen but also the bone marrow representing a site of origin and migration or selective retention of immune subsets and b) how old SPF mice are characterized by a mature but different immune system in comparison to pet shop mice suggesting that current settings for experimental mouse models should be fine-tuned according to their use in pre-clinical mouse research. Understanding how ageing impacts both the innate and adaptive immune cells while distinguishing alterations from physiological pathogen challenges is critical for designing effective vaccines and immunotherapies which could possibly overcome age-dependent refractory diseases. To maximize prospects to translate novel treatments from preclinical to translational research we should further explore the possibilities of improving mouse models by selectively using immune experienced mice to our repertoire of investigative tools while adapting common SPF housing conditions as a more relevant physiological human setting [11, 68, 69].

## Methods

### Mice

In this study, three experimental groups were used: young/middle-aged (6 months) and old (18-20 months) mice kept specific pathogen-free (SPF) and pet shop-supplied mice. For the SPF groups, CD45.1 mice with a C57BL/6 J background were bred in house under SPF conditions at the Charité’s animal housing facility. In all facilities of the Charité’s animal housing, health monitoring is performed regularly according to the guidelines of the Federation of European Laboratory Animal Science Associations (FELASA). Pet shop mice, as a surrogate for wild mice, weighted over 25 g and were purchased from an authorized vendor. All mice were used in accordance with the German law for animal protection with permission of the local authorities.

### Generation of single-cell suspensions of the spleen and bone marrow

Mice were anesthetized with isoflurane followed by cervical dislocation. Spleens and the right hinge were harvested. Single-cell suspensions of the spleen were prepared by smashing each organ through a 70 μm cell strainer. Single-cell suspensions of the bone marrow were prepared into a falcon tube by flushing the shaft using a syringe with a needle. Erythrocytes for both spleen and bone marrow were lysed with 2 ml ACK lysing buffer (Thermo Fisher Scientific) for 4 min at room temperature (RT). Reaction was stopped with 9 ml PBS 0.1% BSA and suspension was passed through a 30 μm cell strainer. After centrifugation (8 min at 310 g at RT) cell pellet was resuspended and incubated for 5 min in 5 ml warm complete culture medium (RPMI containing 10% FBS, 100 U/ml penicillin (Biochrom), 100 mg/ml streptomycin (Biochrom), with 25 U/ml Pierce Universal Nuclease (Thermo Fischer Scientific) in order to reduce viscosity and background from free DNA from any lysed cells. Absolute counts of each suspension were performed on a MACSQuant (Miltenyi Biotec) flow cytometer by acquisition of a defined volume of the cell suspension and counting of CD45^+^, CD19^+^ and CD3^+^ events.

### Staining of surface antigens for mass cytometry and sample acquisition

Staining of surface antigens for mass cytometry was performed as described before [10]. Four new unlabelled carrier protein–free antibodies (abs) were purchased from BD Biosciences (San Jose, CA) or BioLegend (SanDiego, CA) as described below and were conjugated with metal isotopes using Maxpar Ab labeling kits (Fluidigm) according to the manufacturer’s instructions. Metal conjugated abs 162Dy-CXCR4 (clone L276F12, Biolegend), 166Er-CD95 (clone Jo2, BD biosciences), 167Er-CD127 (clone A7R34, Biolegend) and 174Yb-GL7 (clone GL7, Biolegend) were added to the previously established murine surface antigens panel [10]. Cells were acquired on a CyTOF2 mass cytometer upgraded to Helios specifications (CyTOF2/Helios) (Fluidigm). The 0.1 x EQ Four Element Calibration Beads (Fluidigm) were added to the samples for data normalization of the FCS files using the CyTOF software. 330.000 events were recorded in total per file. Tuning was performed each day before measurement following manufacture’s guidelines.

### Polyclonal stimulation and intracellular cytokine staining

A total of 5 × 10^6^ splenocytes per sample in 1 ml complete culture medium (RPMI containing 10% FBS, 100 U/ml penicillin (Biochrom), 100 μg/ml streptomycin (Biochrom), and 50 μM 2-mercaptoethanol (Gibco) were polyclonally stimulated for 5 h with 0.01 mg/ml phorbol-myristateacetate (PMA, Sigma) and 1 mg/ml ionomycin (Sigma), or left untreated, both in the presence of 0.01 mg/ml brefeldin A (Sigma). After incubation, cells were washed twice with PBS and LIVE/DEAD® Fixable Aqua Dead cell stain kit (Thermo Fischer Scientific) was used to enable exclusion of dead cells. Intracellular cytokines IFN-γ (clone: XMG1.2-PE/Cy7, Biolegend), IL-2 (clone: JES6-5H4-APC, Biolegend), IL-4 (11B11-Pe, Biolegend), TNF-α (clone: MP6-XT22-Alexa Fluor 700, Biolegend), IL-10 (clone: JES5-16E3- APC/Cyanine7, Biolegend) CD4 (clone: GK1.5-Brilliant Violet 605, Biolegend), CD8 (clone: 53–6.7-PerCP/Cyanine5.5, Biolegend) and CD40L (clone: MR1-FITC, Invitrogen) staining was performed using fluorochrome-conjugated antibodies for 30 min at 4 °C after fixation and permeabilization with FACS-Lysing and FACS-Perm2 Solution (BD) according to the manufacturer’s protocol. The cells were subsequently analysed using a BD LSR-II cytometer. For CD107α staining, CD107α (clone: 1D4B-efluor 450, eBioscience) was added during stimulation. FACS data were analyzed using FlowJo 9.

### Mass cytometry data analysis

Cytobank [13] was used for initial manual gating and semi-automated SPADE and viSNE to generate spade trees and t-SNE maps accordingly [13–15]. FCS files were pre-processed by manually gating, excluding calibration beads, on cellular events on DNA intercalators, event length, cis-platin and TER119, to remove debris, doublets, normalization beads, live cells and erythrocytes. viSNE analysis was performed on intact, viable cis-platin-, CD45^+^ leukocytes, files were exported and then concatenated using FlowJo 10 visualizing on each tSNE blot same cell event counts. Global lineage 2D t-SNE map and SPADE clustering were generated using CD3, CD11c, CD69, IgM, CD11b, CD19, CD138, CD25, Ly6G, Ly6C, MHCII, PD-L1, CD80, CD86, CD62L, CD40, IgD, CD83, PD-L2, CD8, TCRβ, NK1.1, CD44, CD4, F4/80, CD38 and B220 as clustering markers after gating on single viable intact CD45^+^ leukocytes for all three mouse groups. Based on the median expression of lineage markers CD3, CD11b, TCRβ, CD4, CD8, CD138, CD19, B220 and NK1.1 major immune subsets 1) CD45^+^ CD138^−^ CD3^−^ TCRβ^−^ CD19^−^ innate cells, 2) CD45^+^ CD19^+^ B220^+^ B cells, 3) CD45^+^ CD3^+^ T cells were manually defined. The three major SPADE-clustered subsets 1) innate cells, 2) B cells and 3) T cells were then subjected to viSNE mapping based on lineage-specific differentiation markers.

To generate t-SNE maps for the innate compartment, channels for the following markers were selected to run viSNE: CD11c, CD69, CD11b, CD25, Ly6G, Ly6C, MHCII, PD-L1, CD80, CD86, CD62L, CD40, CD83, PD-L2, CD8, NK1.1, CD44, CD4, F4/80 and CD38. The B cell differentiation viSNE markers included: CD11c, CD69, IgM, CD11b, CD19, CD138, Ly-6C, MHCII, PD-L1, CD80, CD86, CD62L, CD40, IgD, CD83, PD-L2, CD44, F4/80, CD38 and B220. To generate t-SNE maps for the T cell compartment, channels for the following markers were selected to run viSNE: CD11c, CD69, CD11b, CD25, Ly6G, Ly6C, MHCII, PD-L1, CD80, CD86, CD62L, CD83, PD-L2, CD8, TCRβ, NK1.1, CD44, CD4, F4/80 and CD38.

Heatmaps (Figs. 1, 2, 3, 4) were generated using cytobank and show calculated transformed ratio of median marker intensities by table’s minimum within each cell population with global scale range. R, version 3.1, 2017 was used for hierarchical cluster analysis and generating a heat map (Fig. 6b). Spearman correlation distance matrices of population frequencies served as input for Wards agglomerative hierarchical clustering [88]. Heat map coloring was scaled per row, i.e. represents z-scores of population frequencies.

### Statistical analysis

Statistical analysis was performed using Prism (GraphPad Software, SanDiego, CA). Cell numbers and frequencies recovered from spleen and bone marrow were compared using the Mann-Whitney test. *P* values of less than 0.05 were considered significant.

## Supplementary Information

**Additional file 1: Figure S1.** Gating strategy for single live TER119- CD45+ cells. **Figure S2**. Leukocyte composition in spleen and bone marrow ofyoung and old SPF mice. Relative distributions of immune cell subsets identified withinCD45+ leukocytes for spleen and bone marrow (per hinge) respectively. Median subsetproportions are shown for each group as stacked bars (100% CD45). **Figure S3.** Immune populations in the bone marrow of young and old SPF mice. (A) Innate, (B) B, (C) NKT and (D &E) T cell subsets respectively. (B left) Representative gating strategy for developmental stages of single live TER119- CD45+ CD138- CD3- TCRβ- NK1.1- B220+ CD19+ B cells. (C) TCRβ+ and TCRβ- Natural killer T (NKT) cells. (D) Representative histograms of CD8+ T cells depict the expression of the indicated surface molecules on Tnaive, TCM, Teff and TRM respectively. Line indicates mean, ± SD is depicted. **P < 0.01 ***P < 0.001 Mann-Whitney test. NS indicates non-significant changes. **Figure S4.** Immune signatures in the spleen that distinguish young and old SPF mice versus pet shop mice. (A-C) Frequencies of Innate, B and T subsets respectively that are highest in the pet shop mice among all three groups. (D) Frequencies of cell subsets that are lowest in the pet shop mice among all three groups. (E) Frequencies of CD8+ TCm cells among all three groups. Line indicates mean, ± SD is depicted.*P < 0.05, **P < 0.01 ***P < 0.001 Mann-Whitney test. **Figure S5.** Identification of poly-functional CD4+ (A) and CD8+ (B) T cells by Boolean gating. Line indicates mean, ± SD is depicted.*P < 0.05, **P < 0.01 ***P < 0.001 Mann-Whitney test.

## Data Availability

The datasets used and/or analyzed during the current study are available from the corresponding author on reasonable request.
