# Correction to: High-dimensional single cell mass cytometry analysis of the murine hematopoietic system reveals signatures induced by ageing and physiological pathogen challenges

**DOI:** 10.1186/s12979-021-00235-y

**Published:** 2021-05-10

**Authors:** Christos Nikolaou, Kerstin Muehle, Stephan Schlickeiser, Alberto Sada Japp, Nadine Matzmohr, Desiree Kunkel, Marco Frentsch, Andreas Thiel

**Affiliations:** 1 Regenerative Immunology and Aging, BIH Center for Regenerative Therapies (BCRT), Charité Universitätsmedizin Berlin, Berlin, Germany; 2 Institute for Medical Immunology, Charité Universitätsmedizin Berlin, Berlin, Germany; 3 Berlin-Brandenburg School for Regenerative Therapies (BSRT), Charité Universitätsmedizin Berlin, Berlin, Germany; 4 Flow & Mass Cytometry Core Facility, Charité – Universitätsmedizin Berlin and Berlin Institute of Health (BIH), Berlin, Germany

## Abstract

An amendment to this paper has been published and can be accessed via the original article.

**Correction to: Immun Ageing 18, 20 (2021)**

**https://doi.org/10.1186/s12979-021-00230-3**

Following publication of the original article [1], the authors reported an error in Figs. 3, 4 and 5. The following are the error in each figures.

- Figure 3d, young SPF tsne plot has a “B” in the red area.
- Figure 4g, CD4+ TCM analysis has a very small but visible “*” next to the NS
- Figure 5b, IL10 analysis has a very small but visible “*” next to the *

The original article has been corrected.
