# Supplementary material for: High-dimensional single cell mass cytometry analysis of the murine hematopoietic system reveals signatures induced by ageing and physiological pathogen challenges
Source: Immun Ageing. 2021 Apr 20;18:20. doi: 10.1186/s12979-021-00230-3 (PMC8056611; doi:10.1186/s12979-021-00230-3)
Supplement: Supplementary file 1 — Additional file 1: Figure S1. Gating strategy for single live TER119- CD45+ cells. Figure S2. Leukocyte composition in spleen and bone marrow ofyoung and old SPF mice. Relative distributions of immune cell subsets identified withinCD45+ leukocytes for spleen and bone marrow (per hinge) respectively. Median subsetproportions are shown for each group as stacked bars (100% CD45). Figure S3. Immune populations in the bone marrow of young and old SPF mice. (A) Innate, (B) B, (C) NKT and (D &E) T cell subsets respectively. (B left) Representative gating strategy for developmental stages of single live TER119- CD45+ CD138- CD3- TCRβ- NK1.1- B220+ CD19+ B cells. (C) TCRβ+ and TCRβ- Natural killer T (NKT) cells. (D) Representative histograms of CD8+ T cells depict the expression of the indicated surface molecules on Tnaive, TCM, Teff and TRM respectively. Line indicates mean, ± SD is depicted. **P < 0.01 ***P < 0.001 Mann-Whitney test. NS indicates non-significant changes. Figure S4. Immune signatures in the spleen that distinguish young and old SPF mice versus pet shop mice. (A-C) Frequencies of Innate, B and T subsets respectively that are highest in the pet shop mice among all three groups. (D) Frequencies of cell subsets that are lowest in the pet shop mice among all three groups. (E) Frequencies of CD8+ TCm cells among all three groups. Line indicates mean, ± SD is depicted.*P < 0.05, **P < 0.01 ***P < 0.001 Mann-Whitney test. Figure S5. Identification of poly-functional CD4+ (A) and CD8+ (B) T cells by Boolean gating. Line indicates mean, ± SD is depicted.*P < 0.05, **P < 0.01 ***P < 0.001 Mann-Whitney test. [file 12979_2021_230_MOESM1_ESM.pdf]

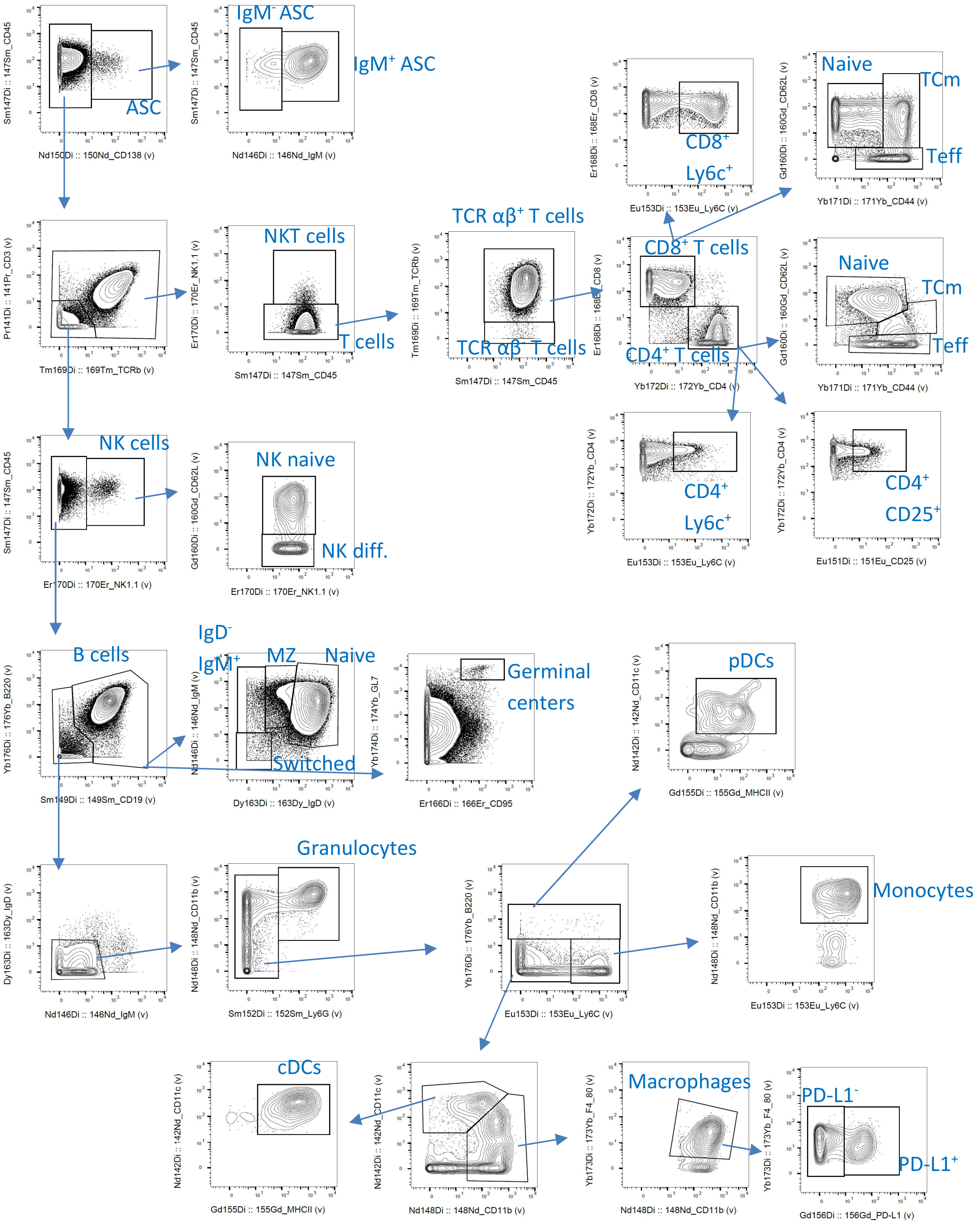

Supplementary Figure 1: Gating strategy for single live TER119^{-} CD45^{+} cells.

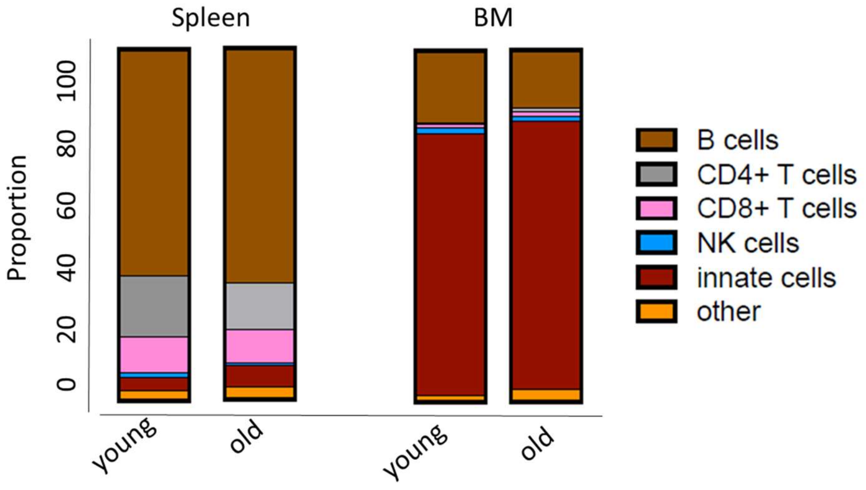

Supplementary Figure 2: Leukocyte composition in spleen and bone marrow of young and old SPF mice. Relative distributions of immune cell subsets identified within CD45+ leukocytes for spleen and bone marrow (per hinge) respectively. Median subset proportions are shown for each group as stacked bars (100% CD45).

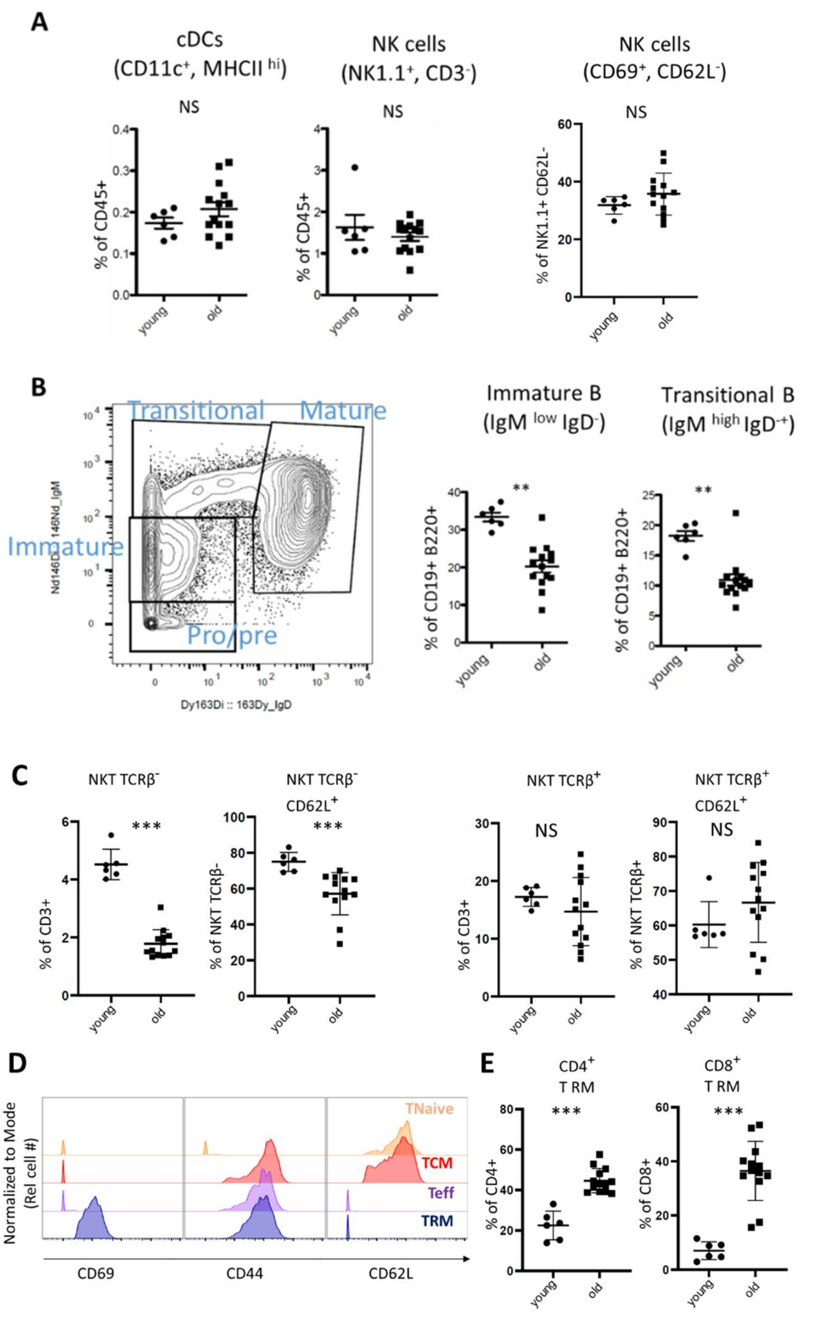

Supplementary Figure 3: **Immune populations in the bone marrow of young and old SPF mice. (A)** Innate, **(B)** B, **(C)** NKT and **(D &E)** T cell subsets respectively. **(B left)** Representative gating strategy for developmental stages of single live TER119$^-$ CD45$^+$ CD138$^-$ CD3$^-$ TCRβ$^-$ NK1.1$^-$ B220$^+$ CD19$^+$ B cells. **(C)** TCRβ$^+$ and TCRβ$^-$ Natural killer T (NKT) cells. **(D)** Representative histograms of CD8$^+$ T cells depict the expression of the indicated surface molecules on Tnaive, TCM, Teff and TRM respectively. Line indicates mean, ± SD is depicted. **P<0.01 ***P<0.001 Mann-Whitney test. NS indicates non-significant changes.

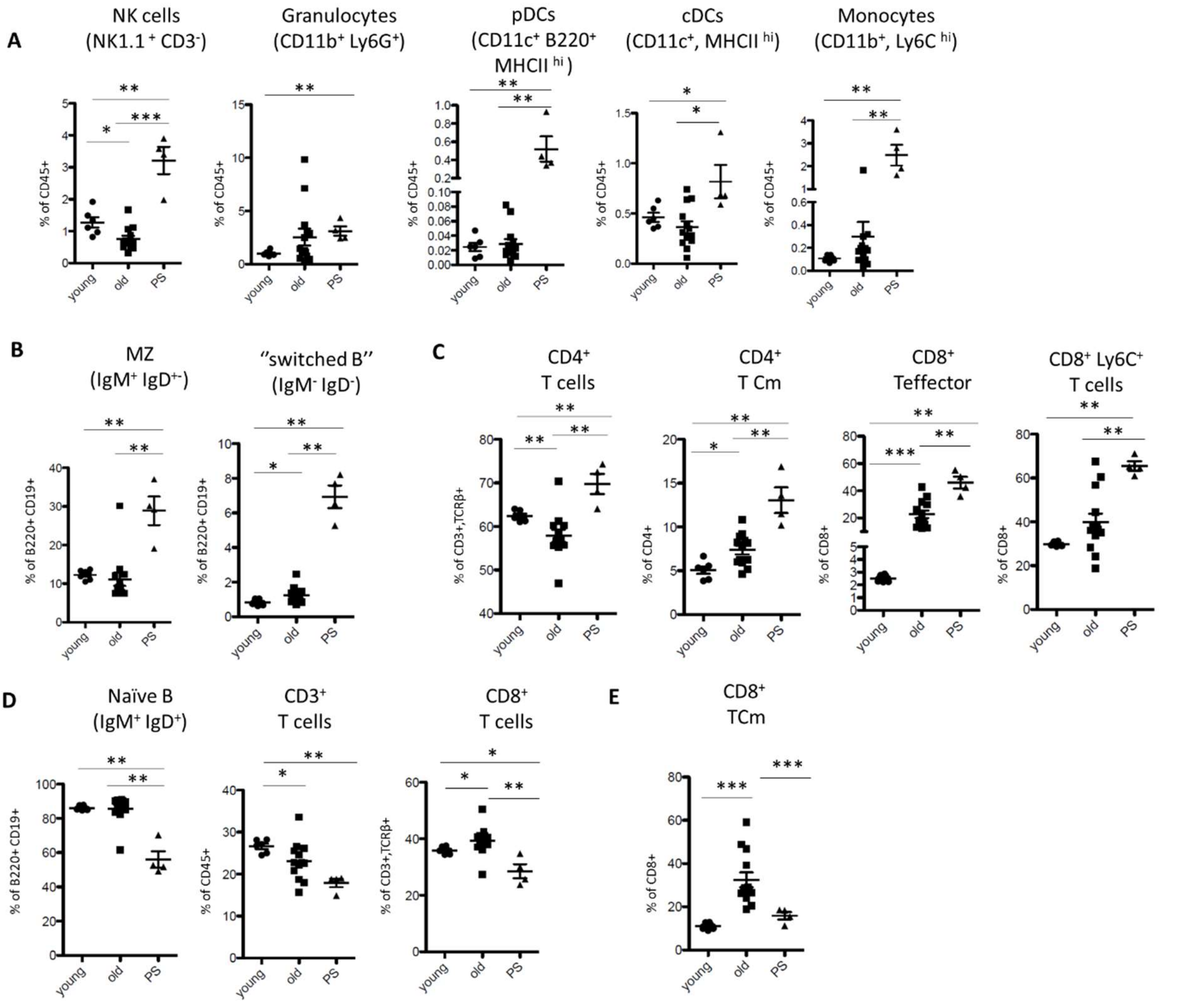

Supplementary Figure 4: Immune signatures in the spleen that distinguish young and old SPF mice versus pet shop mice. (A-C) Frequencies of Innate, B and T subsets respectively that are highest in the pet shop mice among all three groups. **(D)** Frequencies of cell subsets that are lowest in the pet shop mice among all three groups. **(E)** Frequencies of $CD8^+$ TCm cells among all three groups. Line indicates mean, ± SD is depicted.*P<0.05, **P<0.01 ***P<0.001 Mann-Whitney test.

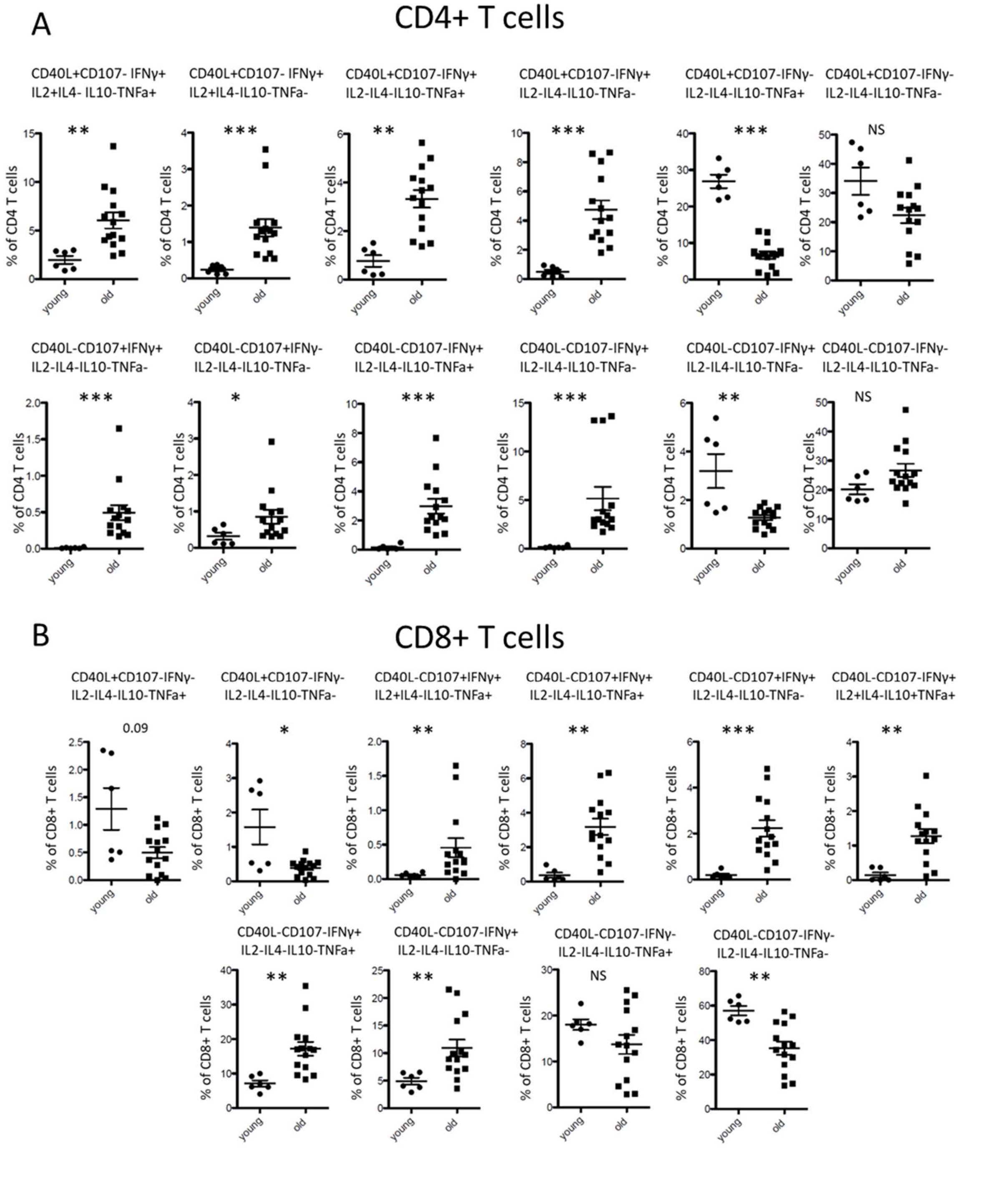

Supplementary Figure 5: Identification of poly-functional CD4+ (A) and CD8+ (B) T cells by Boolean gating. Line indicates mean, ± SD is depicted.*P<0.05, **P<0.01 ***P<0.001 Mann-Whitney test.
